# The Design and Control of a Biomimetic Binocular Cooperative Perception System Inspired by the Eye Gaze Mechanism

**DOI:** 10.3390/biomimetics9020069

**Published:** 2024-01-24

**Authors:** Xufang Qin, Xiaohua Xia, Zhaokai Ge, Yanhao Liu, Pengju Yue

**Affiliations:** 1 Key Laboratory of Road Construction Technology and Equipment of MOE, Chang’an University, Xi’an 710064, China; 2 TianQin Research Center for Gravitational Physics and School of Physics and Astronomy, Sun Yat-sen University (Zhuhai Campus), Zhuhai 519082, China

**Keywords:** gaze mechanism, bionic binocular visual system, binocular cooperative motion

## Abstract

Research on systems that imitate the gaze function of human eyes is valuable for the development of humanoid eye intelligent perception. However, the existing systems have some limitations, including the redundancy of servo motors, a lack of camera position adjustment components, and the absence of interest-point-driven binocular cooperative motion-control strategies. In response to these challenges, a novel biomimetic binocular cooperative perception system (BBCPS) was designed and its control was realized. Inspired by the gaze mechanism of human eyes, we designed a simple and flexible biomimetic binocular cooperative perception device (BBCPD). Based on a dynamic analysis, the BBCPD was assembled according to the principle of symmetrical distribution around the center. This enhances braking performance and reduces operating energy consumption, as evidenced by the simulation results. Moreover, we crafted an initial position calibration technique that allows for the calibration and adjustment of the camera pose and servo motor zero-position, to ensure that the state of the BBCPD matches the subsequent control method. Following this, a control method for the BBCPS was developed, combining interest point detection with a motion-control strategy. Specifically, we propose a binocular interest-point extraction method based on frequency-tuned and template-matching algorithms for perceiving interest points. To move an interest point to a principal point, we present a binocular cooperative motion-control strategy. The rotation angles of servo motors were calculated based on the pixel difference between the principal point and the interest point, and PID-controlled servo motors were driven in parallel. Finally, real experiments validated the control performance of the BBCPS, demonstrating that the gaze error was less than three pixels.

## 1. Introduction

The gaze function is a crucial biological feature of the human visual system. It enables human eyes to identify interest targets in the environment and swiftly shift the gaze to these targets, placing the targets in the foveal region. By doing this, humans can obtain more details and information about the interest targets in the environment and less information about uninteresting regions [1,2,3]. Imitating the gaze function holds great significance for the advancement of fields such as human–robot interaction [4,5], autonomous driving [6], virtual reality [7], etc. Moreover, imitating the gaze function for the humanoid eye perception system has the potential to filter redundant information from huge data, optimize the use of computing and storage resources, enhance scene comprehension, and improve perception accuracy. This imitation stands as an important step in advancing humanoid eye intelligent perception.

The primary work in imitating the eye gaze function is to research the eye gaze mechanism. The mechanism of eye movement, which plays a key role in the gaze function, has been widely studied. Marg introduced electro-oculography (EOG) as a method for measuring eye movement by obtaining eye potentials through electrodes around the eyes [8]. However, this contact measurement method suffers from lower precision and poor portability. Subsequently, the presentation of video-oculography (VOG) offered a more accurate and portable non-contact eye movement measurement method [9]. VOG used cameras mounted on wearable devices to capture the position of the pupil. Nevertheless, this method lacked a stimulus-presenting monitor and recording device, preventing independent measurements. The design of the all-in-one eye-movement-measuring device overcame this limitation, enabling independent and accurate eye movement measurement [10]. Through the gradual improvement of eye-movement-measurement devices, the factors affecting the eye movement mechanism have been studied [11,12,13,14]. Those studies have found that factors such as gender differences [12], cross-cultural differences in facial features [13], and stimulus contrast and spatial position differences [14] contribute to saccadic pattern differences. Now, many researchers are interested applying our increasingly robust understanding of the eye movement mechanism to the structural design and motion control of humanoid eye perception systems, which can imitate the gaze function.

In this structural design field, the methods can be divided into two categories. The first method refers to imitating the physiological structure of the extraocular muscles. This usually involves using a spherical link structure [15], spherical parallel mechanism [16], or multiple flexible ropes parallel mechanism [17] to design a device that achieves a highly realistic imitation of the physiological structure of the human eye. However, researchers face difficulties in reducing the sizes of these devices. With that goal, some studies have proposed that super-coiled polymers (SCPs) [18] or pneumatic artificial muscles (PAMs) [19] can be used to replace rigid materials or ropes in the design of a device. However, achieving precise control of these devices has remained challenging. The second method is to imitate the effect of the actual motion of the eye. This method usually uses the servo motor as the power source, which can reduce difficulty of controlling the device. Fan et al. [20] designed a bionic eye that could tilt, pan, and roll with six servo motors. However, a rolling motion is generally not required for a device that imitates the gaze function [21]. Some studies have focused on the design of devices that can tilt and pan with four servo motors [22,23]. However, those four servo motors are not synced for the cooperative motion of human eyes. Thus, a device was designed that could tilt and pan with three servo motors [24]. However, this device lacked a camera position adjustment component. It would have been difficult to ensure that the vertical visual field of the two cameras was consistent due to the potential for assembly errors, which affect gaze accuracy. Moreover, in a dynamic analysis [25,26] of this device, it was found that the torque of the servo motor responsible for tilting exceeded that of the other two servo motors, impacting the overall performance and efficiency. Therefore, further optimization of the structure is needed.

In regard to motion control, researchers have explored two distinct approaches. One approach is a motion-control strategy driven by a set target [22,27,28,29,30,31]. Mao et al. [28] proposed a control method that could be described as a two-level hierarchical system. This method could imitate the horizontal saccade of human eyes. Subsequently, a control method [30] was designed that employed a hierarchical neural network model based on predictive coding/biased competition with divisive input modulation (PC/BC-DIM) to achieve gaze shifts in 3D space. Despite the effect of the control method being consistent with that of human eye movement, the neural network requires plenty of data support compared to traditional algorithms. As an alternative, a traditional control algorithm based on 3D or 2D information of a target has raised attention [22,31,32]. For example, a vision servo based on 2D images was proposed to control the pose of each camera and achieve the fixation of a target [22]. Rubies et al. [32] calibrated the relationship between the eye positions on a robot’s iconic face displayed on a flat screen and the 3D coordinates of a target point, thereby controlling the gaze toward the target. The motion-control strategy driven by the set target has clear objectives and facilitates precise motion control. However, it falls short in imitating the spontaneous gaze behavior of humans and may ignore other key information, which leaves it impossible to fully understand the scene. Another approach is a motion-control strategy driven by a salient point, which can make up for these shortages. Researchers have made significant progress in saliency-detection algorithms, including classical algorithms [33,34,35] and deep neural networks [36,37,38]. The results of saliency detection are increasingly aligning with human eye selective attention. Building on this foundation, Zhu et al. introduced a saccade control strategy driven by binocular attention based on information maximization (BAIM) and a convergence control strategy based on a two-layer neural network [39]. However, this method cannot simultaneously execute saccade control and convergence control to imitate the cooperative motion of human eyes.

Recognizing the limitations of the above work, we have proposed a design and control method of the BBCPS inspired by the gaze mechanism of human eyes. To address the issues of servo motor redundancy and the lack of camera position adjustment components in the existing systems, a simple and flexible BBCPD was designed. The BBCPD consisted of RGB cameras, servo motors, pose adjustment modules, braced frames, calibration objects, transmission frames, and bases. It was assembled according to the innovative principle of symmetrical distribution around the center. A simulation demonstrated that the BBCPD achieved a great reduction in energy consumption and an enhancement in braking response performance. Furthermore, we developed an initial position calibration technique to ensure that the state of the BBCPD could meet the requirement of the subsequent control method. On this basis, we propose a control method of the BBCPS, aiming to fill the gap in binocular cooperative motion-control strategies, driven by interest points in the existing systems. In the proposed control method, a PID controller is introduced to realize precise control of a single servo motor. A binocular interest-point extraction method based on frequency-tuned and template-matching algorithms is presented to identify interest points. A binocular cooperative motion-control strategy is then outlined to coordinate the motion of servo motors in order to move the interest point to the principal point. Finally, we summarize the results of real experiments, which proved that the control method of the BBCPS could control the gaze error within three pixels.

The main contributions of our work are as follows. (a) We designed and controlled the BBCPS to simulate the human eye gaze function. This contributes to deepening our understanding of human eye gaze mechanisms and advancing the field of humanoid eye intelligent perception. (b) Our designed BBCPD features a simple structure, flexibility and adjustability, low energy consumption, and excellent braking performance. (c) We developed an interest-point-driven binocular cooperative motion-control method, perfecting the research on the control strategy for imitating human eye gaze. Additionally, we calibrated the initial position of the BBCPS via our self-developed calibration technique. This eliminates the need for repeated calibration in subsequent applications, improving the operational convenience of the BBCPS. What’s more, our proposed binocular interest-point extraction method based on frequency-tuned and template-matching algorithms enriches the current research in the field of salient point detection.

## 2. Gaze Mechanisms of Human Eyes

**Figure 1:**
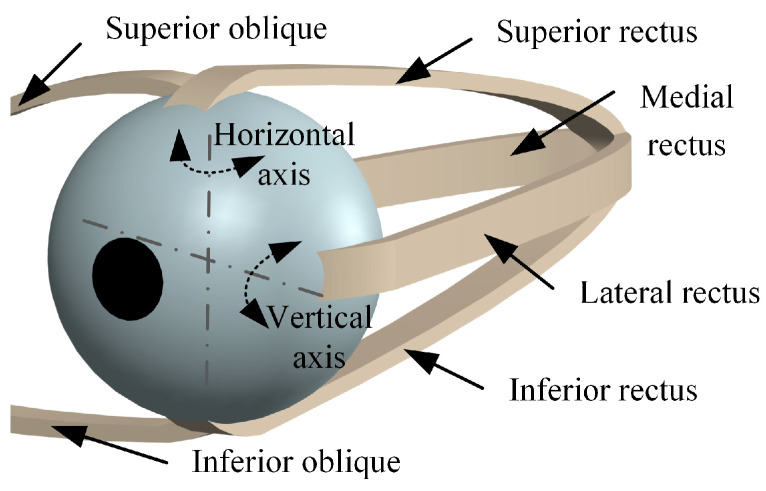
Eyeball movement.

The movement of the eyeball is crucial to the gaze function. As shown in Figure 1, the eyeball is usually regarded as a perfect sphere, and its movement is controlled by the medial rectus, lateral rectus, superior rectus, inferior rectus, superior oblique, and inferior oblique [40,41,42]. These muscles contract and relax to perform different eye movements. The superior and inferior oblique muscles assist in the torsional movement of the eyeball. Torsional eye movements, characterized by minimal overall variability (approximately 0.10°), are an unconscious reflex and strictly physiologically controlled [21]. The superior and inferior rectus muscles rotate the eyeball around the horizontal axis, and the lateral and medial rectus muscles allow the eyeball to rotate around the vertical axis.

Vertical and horizontal movements of the eyeball are important for the line-of-sight shift in the gaze function [43], which refers to the process of shifting the current line of sight to the interest point through eyeball movements during visual observation. This process involves saccade and convergence. Saccade is a conjugate movement that can achieve the line-of-sight shift of human eyes in both horizontal and vertical directions. Convergence describes a non-conjugate movement of human eyes in the horizontal direction, where the two eyes move in opposite directions to help humans observe points at different depths. By coordinating saccade and convergence, the two eyes can shift their line of sight to any point of interest in three-dimensional space.

**Figure 2:**
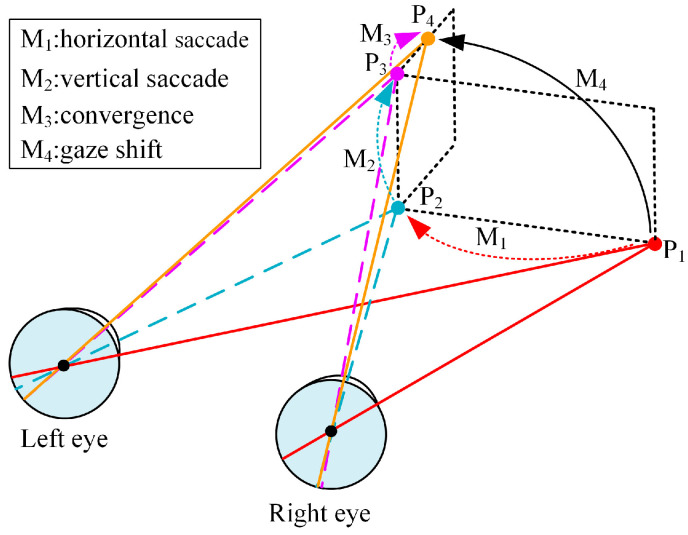
Human eye cooperative movement.

To better understand the movement mechanism of the gaze function, we have created a schematic diagram of the human eye cooperative movement, shown in Figure 2. From a physiological point of view, the human eye changes from the gaze point $P_{1}$ to the gaze point $P_{4}$ through the coordination of saccade and convergence. We assume that the eye movement is sequential, and the process $M_{4}$ is decomposable into $M_{1}$, $M_{2}$, and $M_{3}$. Specifically, the shift from the gaze point $P_{1}$ to the gaze point $P_{2}$ is first achieved through the horizontal saccadic movement $M_{1}$. The shift from the gaze point $P_{2}$ to the gaze point $P_{3}$ is then accomplished through the vertical saccadic movement $M_{2}$. In the end, the convergent movement $M_{3}$ is employed to shift the gaze point $P_{3}$ to the gaze point $P_{4}$.

## 3. Structural Design

**Figure 3:**
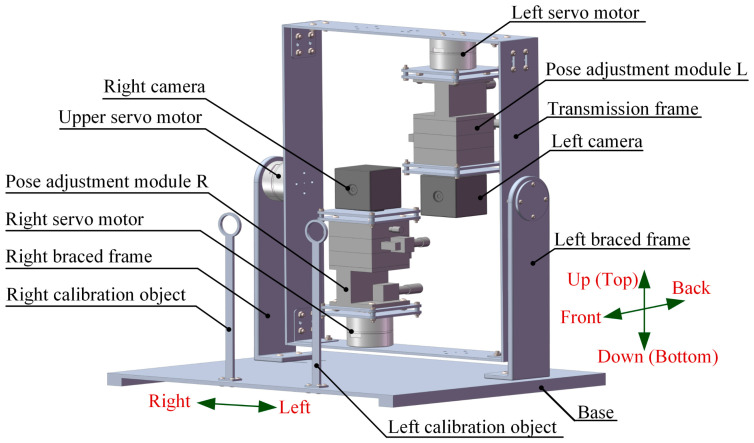
Three-dimensional model of the BBCPD.

Inspired by the gaze mechanism of human eyes, the mechanical structure of the BBCPD was designed. Its 3D model is shown in Figure 3. The device is composed of two RGB cameras, three servo motors, two pose adjustment modules, two braced frames, two calibration objects, a transmission frame, and a base. RGB cameras capture images, and servo motors act as the power source. Pose adjustment modules are used to accurately adjust the pose of cameras toward different desired locations. This indicates an increased flexibility of the BBCPD. The transmission frame is designed to transmit motion. Calibration objects are used to calibrate the initial position of the BBCPD. Braced frames serve to ensure the suspension of the transmission frame, guaranteeing the normal operation of the upper servo motor. The role of the base is to ensure the stable operation of the BBCPD.

In the component design, the transmission frame and the base are designed as parallel symmetrical structures motivated by the stability of the symmetrical structure. The braced frame adopts an L-shaped structure because this is highly stable and its different ends can be used to connect various other components. The design inspiration for the pose adjustment module is derived from the screw motion mechanism and the turbine worm drive. Based on the former, the camera can be adjusted in three directions: front–back, left–right, and up–down. Simultaneously, inspired by the turbine worm drive to change the direction of rotation, the roll, pan, and tilt adjustment of the camera are skillfully realized. The top of the calibration object is designed as a thin-walled ring, as the circle center is easy to detect, facilitating the subsequent zero-position adjustment of the servo motor. Furthermore, lightweight and high-strength aluminum alloy is selected as the component material.

During the assembly of the components, the principle of symmetrical distribution about the center of the transmission frame is followed, though it loses some of its biomimetic morphology compared to the classic principle of a symmetrical low center of gravity [2]. We recognize that the torque required of the upper servo motor in the BBCPD is markedly smaller than that in a system assembled according to the symmetrical low center of gravity principle. This means that the power consumption is lower in the BBCPD. In addition, the rotational inertia of the upper servo motor load around the rotation axis in the BBCPD is smaller than that in the system assembled according to the symmetrical low center of gravity principle. The BBCPD also has better braking performance. The detailed reason will be explained when we present our subsequent dynamic analysis of the upper servo motor.

**Figure 4:**
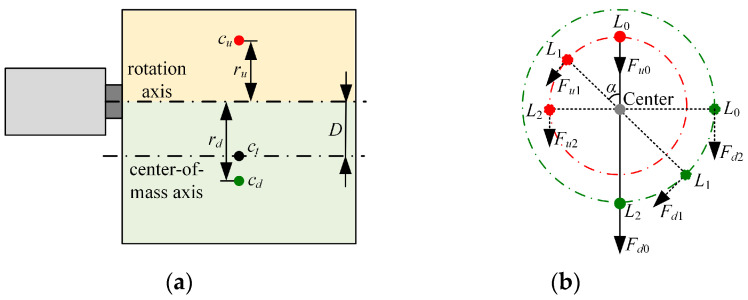
Dynamic analysis. (**a**) Structural sketch of the upper servo motor and its load, (**b**) force analysis of the upper servo motor.

In Figure 4, $c_{t}$ is the center of mass of the whole load, and D represents the vertical distance from $c_{t}$ to the rotation axis. The rotation axis serves as the boundary, and the load is divided into upper and lower parts. The center of mass of the upper part is denoted as $c_{u}$, and the vertical distance from $c_{u}$ to the rotation axis is defined as $r_{u}$. The symbol $c_{d}$ is the center of mass of the lower part, and the vertical distance from $c_{d}$ to the rotation axis is expressed as $r_{d}$. $L_{i}$ (*i* = 0, 1, …) represents the location of the motor, and $\theta_{i}$ denotes the rotation angle of the motor at $L_{i}$. The mass of the upper part is represented as $m_{u}$, $m_{d}$ represents the mass of the lower part, and *g* means the acceleration of gravity.

According to the parallel-axis theorem, the rotational inertia of the whole load around the rotation axis J is given by Equation (1).

(1) $$J=J_{c}+mD^{2},$$

where *m* represents the mass of the load of the upper servo motor, and $J_{c}$ is the rotational inertia of the whole load around the center-of-mass axis.

Next, we conducted the force analysis on the motor and derived the torque of the load on the motor at $L_{i}$.

(2) $$M_{i}=F_{ui}r_{u}-F_{di}r_{d}=m_{u}g\sin\theta_{i}r_{u}-m_{d}g\sin\theta_{i}r_{d}.$$

Thus, the torque of the motor at $L_{i}$ could be obtained by combining Equations (1) and (2).

(3) $$T_{i}=J\omega -M_{i}=(J_{c}+mD^{2})\omega -(m_{u}g\sin\theta_{i}r_{u}-m_{d}g\sin\theta_{i}r_{d}),$$

where ω is the angular acceleration of the motor. According to Equation (3), it can be observed that as D and the difference between $m_{u}$ and $m_{d}$ decrease, $T_{i}$ and J become smaller. In the BBCPD, D and the difference between $m_{u}$ and $m_{d}$ are close to 0. However, in the system assembled according to the symmetrical low center of gravity principle, both D and the difference between $m_{u}$ and $m_{d}$ are greater than 0.

Afterward, we performed stress–strain analyses on the BBCPD using software and refined the dimensions of the components. The initial position of the BBCPD was defined as the state that imitates the approximately symmetrical distribution of eyeballs about the midline of the face when humans gaze at infinity. In other words, the camera optical center coincides with the rotation center, and the cameras are parallel to each other. Ultimately, the layout of each component in the space is introduced in the order from left to right.

The bottom of the left servo motor is installed at the bottom of the transmission frame, 64 mm to the left side of the transmission frame. Its shaft end is connected to the bottom of the pose adjustment module L, and the top of the pose adjustment module L is linked to the bottom of the left camera. The optical center of the left camera passes through the shaft of the left servo motor. The bottom of the right servo motor is installed at the top of the transmission frame, 64 mm to the right side of the transmission frame. Its shaft end is connected to the bottom of the pose adjustment module R, and the top of the pose adjustment module R is linked to the base of the right camera. The optical center of the right camera passes the shaft of the right servo motor. The shaft end of the upper servo motor is connected to the right side of the transmission frame, 180 mm above the bottom of the transmission frame, and the shaft passes through the optical centers of two cameras. The bottom of the upper servo motor is connected to the top of the braced frame. Braced frames are installed on the left and right sides of the base in opposite poses to make full use of the space. The left and right calibration objects are vertically fixed to the front of the base, located at 125 mm and 245 mm on the left side of the base, respectively. The plane they lie on is parallel to the planes containing the shafts of the three servo motors.

The BBCPD with three degrees of freedom can effectively imitate the cooperative motion of human eyes. Specifically, the left servo motor and the right servo motor drive the left camera and the right camera to pan, respectively, thereby realizing the imitation of horizontal saccade and convergence. The upper servo motor drives the left camera and the right camera to tilt simultaneously through the transmission frame to imitate the vertical saccade. The design fully considers the human eye gaze mechanism and provides hardware support for imitating the gaze function of human eyes.

## 4. Initial Position Calibration

The initial position calibration of the BBCPD is a crucial step for achieving the control of the BBCPS. This is designed so that the initial position of the BBCPD meets the requirements of the subsequent control method that the camera optical center coincides with the rotation center and the cameras are parallel to each other. In addition, once the initial position is determined, the BBCPD does not need to be recalibrated during subsequent applications, saving time and resources. In the design of the BBCPD, we default that the initial position of the BBCPD is in line with the requirement of the subsequent control method. However, due to inevitable errors during the manufacturing and assembly processes, the initial position of the real BBCPD makes it challenging to guarantee this requirement is met. Additionally, the zero-positions of servo motors may not be set at the ideal initial position for the real BBCPD. Therefore, we provided the initial position calibration technology of the BBCPD to determine the initial position by calibrating and adjusting the camera poses and the zero-positions of servo motors.

### 4.1. Camera Pose Calibration and Adjustment

Camera pose calibration and adjustment refers to calibrating the rotation and translation parameters from the base coordinate frame to the camera coordinate frame, and then changing the camera pose using the pose adjustment module. The camera coordinate frame is a right-handed coordinate frame with the optical center as the origin and straight lines parallel to the length and width of the photosensitive plane as the horizontal axis and the vertical axis. The base coordinate frame is defined as a right-handed coordinate frame with the rotation center as the origin point, the horizontal rotation axis as the horizontal axis, and the vertical rotation axis as the vertical axis.

**Figure 5:**
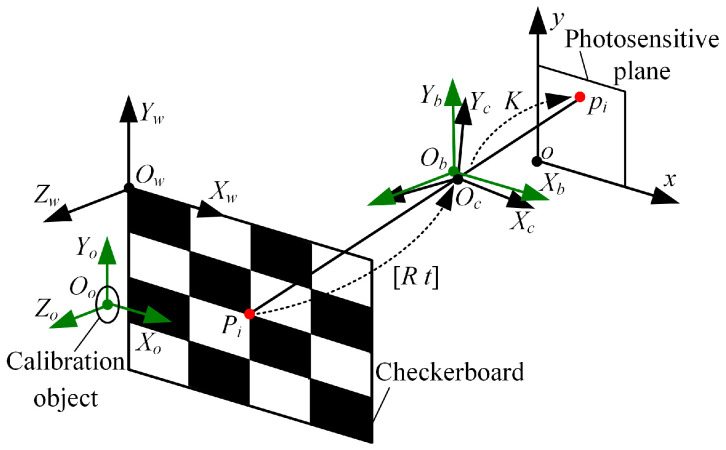
Calibration principle of the rotation parameters.

First, we devoted our time to calibrating the rotation parameters, in order to adjust the camera poses to achieve mutual parallelism of the photosensitive planes of the two cameras. Taking the left camera as an example, under corrected lens distortion, the calibration principle of the rotation parameters is shown in Figure 5. The camera coordinate frame is denoted as $O_{c}X_{c}Y_{c}Z_{c}$. The base coordinate frame is represented by $O_{b}X_{b}Y_{b}Z_{b}$, and the calibration object coordinate frame is described by $O_{o}X_{o}Y_{o}Z_{o}$. The original point $O_{o}$ is the ring center of the left calibration object, and the plane $O_{o}X_{o}Y_{o}$ is the vertical center plane of the ring. $O_{w}X_{w}Y_{w}Z_{w}$ is the world coordinate frame. The original point $O_{w}$ is set at the upper-right corner of the checkerboard. The horizontal and vertical directions of the checkerboard are the directions of the axis $X_{w}$ and the axis $Y_{w}$, respectively.

The checkerboard is placed against the calibration objects so that the plane $O_{w}X_{w}Y_{w}$ is parallel to the plane $O_{o}X_{o}Y_{o}$. In the real BBCPD, the plane $O_{o}X_{o}Y_{o}$ is parallel to the plane $O_{b}X_{b}Y_{b}$. Thus, by deriving the rotation relationship between the coordinate frame $O_{w}X_{w}Y_{w}Z_{w}$ and the coordinate frame $O_{c}X_{c}Y_{c}Z_{c}$, the rotation parameters can be calibrated. According to the linear projection principle [44], the relationship between the 3D corner point $P_{i}$ and the 2D corner point $p_{i}$ is expressed via Equation (4).

(4) $$p_{i}=sK\left[\begin{array}{cc} R & t \end{array}\right]P_{i},$$

where K is the intrinsic matrix, s is the scale factor, R is the rotation matrix, and t is the translation matrix. We use all corners of the checkerboard to solve R with known $P_{i}$, $p_{i}$, and K. Based on Equation (4), Equation (5) is obtained, and then R is calculated using the least-squares method.

(5) $$K^{-1}\left[p_{0},\;p_{1},\;\cdots ,\;p_{n}\right]=s\left[\begin{array}{cc} R & t \end{array}\right]\left[P_{0},\;P_{1},\;\cdots ,\;P_{n}\right],$$

where *n* (*n* > 4) is the number of corners. Due to the small errors caused by the manufacturing and assembly processes in normal cases, the absolute values of the parameters $\theta_{x}$, $\theta_{y}$, and $\theta_{z}$ are generally no more than 90°. The rotation parameters $\theta_{x}$, $\theta_{y}$, and $\theta_{z}$ can be uniquely calculated by using Equation (6) based on the rotation order of $Z_{w}Y_{w}X_{w}$.

(6) $$\left\{\begin{array}{c} \theta_{x}=\arctan\frac{-R_{23}}{R_{33}}\\ \theta_{y}=\arctan\frac{R_{13}}{-\sin\theta_{x}R_{23}+\cos\theta_{x}R_{33}}\\ \theta_{z}=\arctan\frac{\cos\theta_{x}R_{21}+\sin\theta_{x}R_{31}}{\cos\theta_{x}R_{22}+\sin\theta_{x}R_{32}} \end{array}\right.,\;\mathrm{with}\;R^{-1}=\left[\begin{array}{ccc} R_{11} & R_{12} & R_{13}\\ R_{21} & R_{22} & R_{23}\\ R_{31} & R_{32} & R_{33} \end{array}\right].$$

According to the above principle, the rotation parameters of the right camera can be solved. The poses of the left and right cameras are changed by sequentially adjusting the tilting, panning, and rolling angles of the pose adjustment module L and the pose adjustment module R. The tilting, panning, and rolling angles are the corresponding negative rotation parameters. At this time, the camera coordinate frame is parallel to the base coordinate frame, and the photosensitive planes are parallel to each other.

**Figure 6:**
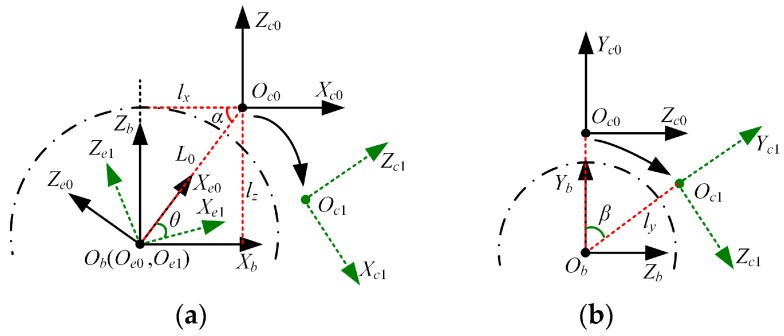
Calibration principle of the translation parameters. (**a**) Translation parameters in $X_{b}$ and $Z_{b}$ directions, (**b**) translation parameters in $Y_{b}$ direction.

Next, we calibrated the translation parameters to adjust the camera poses so that the camera optical center coincided with the rotation center [45]. Based on the above steps, the calibration principle of the translation parameters, taking the left camera as an example, is depicted in Figure 6. The camera coordinate frame at the initial position $O_{c0}X_{c0}Y_{c0}Z_{c0}$ is parallel to the base coordinate frame $O_{b}X_{b}Y_{b}Z_{b}$. $O_{c1}X_{c1}Y_{c1}Z_{c1}$ represents the camera coordinate frame after motion. The end-of-motion coordinate frame at the initial position $O_{e0}X_{e0}Y_{e0}Z_{e0}$ is established by taking the line connecting the point $O_{c0}$ and the point $O_{b}$ as the $X_{e0}$-axis, the $Y_{b}$-axis as the $Y_{e0}$-axis, and the point $O_{b}$ as the origin. The end-of-motion coordinate frame after motion is denoted as $O_{e1}X_{e1}Y_{e1}Z_{e1}$. The rotation angles of the camera around the $Y_{b}$-axis and $X_{b}$-axis are θ and β. The translation parameters in $X_{b}$, $Y_{b}$, and $Z_{b}$ are denoted as $l_{x}$, $l_{y}$, and $l_{z}$.

Let $T_{ij}$ represent the transformation from the coordinate frame $O_{i}X_{i}Y_{i}Z_{i}$ to the coordinate frame $O_{j}X_{j}Y_{j}Z_{j}$. Based on the transformation relationship between the coordinate frame $O_{c0}X_{c0}Y_{c0}Z_{c0}$ and the coordinate frame $O_{c1}X_{c1}Y_{c1}Z_{c1}$ in Figure 6a, Equation (7) can be obtained.

(7) $$T_{c0}^{c1}=T_{e1}^{c1}T_{e0}^{e1}T_{c0}^{e0},$$

where the expressions of $T_{c0}^{c1}$, $T_{e1}^{c1}$, $T_{e0}^{e1}$, and $T_{c0}^{e0}$ are in Equation (8).

(8) $$\begin{array}{l} T_{c0}^{c1}=\left[\begin{array}{cccc} r_{11} & r_{12} & r_{13} & t_{1}\\ r_{21} & r_{22} & r_{23} & t_{2}\\ r_{31} & r_{32} & r_{33} & t_{3}\\ 0 & 0 & 0 & 1 \end{array}\right],T_{e1}^{c1}=\left[\begin{array}{cccc} \cos\alpha & 0 & -\sin\alpha & L_{0}\sin\alpha \\ 0 & 1 & 0 & 0\\ \sin\alpha & 0 & \cos\alpha & -L_{0}\cos\alpha \\ 0 & 0 & 0 & 1 \end{array}\right],\\ T_{e0}^{e1}=\left[\begin{array}{cccc} \cos\theta & 0 & -\sin\theta & 0\\ 0 & 1 & 0 & 0\\ \sin\theta & 0 & \cos\theta & 0\\ 0 & 0 & 0 & 1 \end{array}\right],\;\mathrm{and}\;T_{c0}^{e0}=\left[\begin{array}{cccc} \cos\alpha & 0 & \sin\alpha & 0\\ 0 & 1 & 0 & 0\\ -\sin\alpha & 0 & \cos\alpha & L_{0}\\ 0 & 0 & 0 & 1 \end{array}\right]. \end{array}$$

Equation (9) can be obtained by joining Equations (7) and (8).

(9) $$\cos\alpha ={\left(M^{2}+1\right)}^{-\frac{1}{2}},\;\sin\alpha =\sqrt{\frac{M^{2}}{M^{2}+1}},\;\mathrm{and}\;L_{0}=\frac{t_{1}}{\sin\alpha -\cos\alpha \sin\theta -\sin\alpha \cos\theta },$$

where $M=\frac{-t_{3}\sin\theta -t_{1}\cos\theta +t_{1}}{-t_{1}\sin\theta +t_{3}\cos\theta -t_{3}}.$ $T_{c0}^{\;c1}$ can be solved using Equation (4), and θ is the known rotation angle. Therefore, $l_{x}$ and $l_{z}$ can be calculated using Equation (10).

(10) $$l_{x}=L_{0}\cos\alpha ,\;\mathrm{and}\;l_{z}=L_{0}\sin\alpha .$$

According to the above principle, partial translation parameters of the left and right cameras can be solved. The pose adjustment module L and the pose adjustment module R are adjusted in the left–right and front–back directions according to the corresponding negative calculated translation parameters. Upon completion of the adjustments, the remaining translation parameters in the vertical direction continue to be calibrated, which is similar to the principle of calibrating $l_{x}$ and $l_{z}$.

We also take the left camera as an example. Based on the transformation relationship between the coordinate frame $O_{c0}X_{c0}Y_{c0}Z_{c0}$ and the coordinate frame $O_{c1}X_{c1}Y_{c1}Z_{c1}$ in Figure 6b, Equation (11) can be obtained.

(11) $$T_{c0}^{c1}=T_{b}^{c1}T_{c0}^{b}=\left[\begin{array}{cccc} 1 & 0 & 0 & 0\\ 0 & \cos\beta & \sin\beta & -l_{y}\\ 0 & -\sin\beta & \cos\beta & 0\\ 0 & 0 & 0 & 1 \end{array}\right]\left[\begin{array}{cccc} 1 & 0 & 0 & 0\\ 0 & 1 & 0 & l_{y}\\ 0 & 0 & 1 & 0\\ 0 & 0 & 0 & 1 \end{array}\right]=\left[\begin{array}{cccc} 1 & 0 & 0 & 0\\ 0 & \cos\beta & \sin\beta & \cos\beta l_{y}-l_{y}\\ 0 & -\sin\beta & \cos\beta & -\sin\beta l_{y}\\ 0 & 0 & 0 & 1 \end{array}\right].$$

Therefore, the translation parameter $l_{y}$ in the $Y_{b}$ direction is

(12) $$l_{y}=\frac{-t_{3}}{\sin\beta },$$

where $t_{3}$ is the translation component in the $Z_{c1}$ direction of $T_{c0}^{c1}$, which can be solved using Equation (4). The translation parameters of the left and right cameras in the vertical direction are solved. Once the camera pose is adjusted through the adjustment module, it reaches the state where the optical center coincides with the rotation center.

### 4.2. Servo Motor Zero-Position Calibration and Adjustment

The calibration and adjustment of the zero-position of the servo motor refers to calculating the angle at which the zero-position of the servo motor rotates to the initial position, and then resetting the zero-position of the servo motor. This can ensure that the BBCPS returns to the initial position no matter what movement it performs.

**Figure 7:**
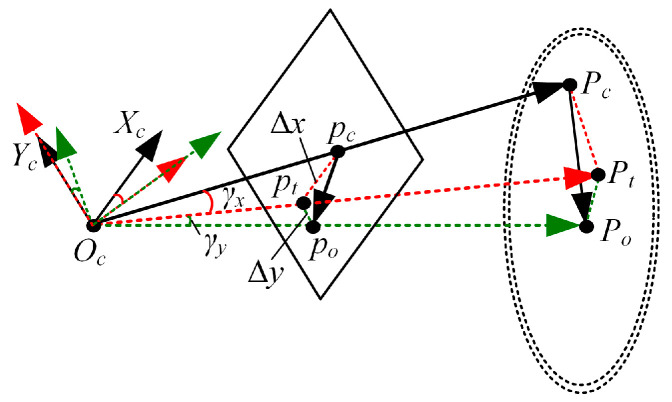
Calibration principle of the angle.

Considering that the optical center coincides with the rotation center, the calibration principle of the angle for the left and upper motors is shown in Figure 7. The projection point of the ring center of the left calibration object in the left camera is $p_{0}$ when the servo motors are at their zero-position. The projection point of the ring center of the left calibration object in the left camera is $p_{c}$ when the servo motors are in the initial position of the BBCPS. The projection point of the ring center of the left calibration object in the left camera is $p_{t}$ when the angle of the upper servo motor is $\gamma_{y}$. The horizontal difference between $p_{c}$ and $p_{0}$ is represented by ∆x, and the vertical difference is ∆y. According to the equal vertex angle theorem, the angle of the left servo motor $\gamma_{x}$ and the angle of the upper servo motor $\gamma_{y}$ can be calculated using Equation (13).

(13) $$\gamma_{x}=\arctan\left|\frac{\Delta x}{f}\right|,\;\mathrm{and}\;\gamma_{y}=\arctan\left|\frac{\Delta y}{f}\right|,$$

where *f* is the focal length of the camera. In addition, the rotation direction of the left servo motor is defined as positive rotation when ∆x is positive. The definition of the direction of the upper servo motor is the same as the left servo motor.

**Figure 8:**
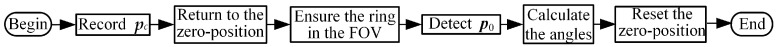
Procedure of the zero-position of the servo motor calibration and adjustment.

However, Equation (13) will fail when $\gamma_{x}$ or $\gamma_{y}$ is greater than a certain angle. When the servo motors are in their zero-position, the ring center may not be detected in the image because the ring center is beyond the field of view (FOV) of the left camera. Considering the above situation, we developed the procedure of the zero-position of the servo motor calibration and adjustment as shown in Figure 8. To prevent multiple circles from being detected, the background of the ring in the image should be kept simple, such as a pure-color wall. First, $p_{c}$ is determined using the Hough circle detection algorithm [46] and recorded as the target point. Next, the left and upper servo motors are returned to their zero-position. To ensure the ring in the FOV, the left servo motor is required to rotate positively *n* and the upper servo motor needs to rotate positively *m*. The values of *n* and *m* are from 0° to 360°. Subsequently, $p_{0}$ can be detected using the Hough circle detection algorithm and the angles $\gamma_{x}$ and $\gamma_{y}$ can be calculated. The angles at which the left and upper servo motors rotate to the initial position are *n +* $\gamma_{x}$ and *m +* $\gamma_{y}$, respectively. Finally, the zero-positions of the left and upper servo motors are reset. The zero-position of the right servo motor can also be reset according to the above procedure.

## 5. Control Method

In this section, the gaze mechanism of human eyes is imitated from the perspective of control. First, in our study, the motion-control method of a single servo motor based on a PID controller was introduced. Then, we developed a binocular interest-point extraction method based on frequency-tuned and template-matching algorithms. Furthermore, we proposed a binocular cooperative motion strategy to move the interest point to the principal point. Finally, real experiments were conducted to verify the effectiveness of the control method.

### 5.1. Motion Control of a Single Servo Motor

A PID controller is widely used in servo motor control because of its relative simplicity, easy adjustment, and fair performance [47]. The control principle is shown in Equation (14).

(14) $$u(t)=K_{P}\cdot e(t)+K_{i}\int_{0}^{t}e(t)dt+K_{d}\frac{de(t)}{dt},$$

where $K_{p}$ is the proportional gain, $K_{i}$ is the integral gain, $K_{d}$ is the differential gain, *t* is the time, and e(t) is the error.

By tuning $K_{p}$, $K_{i}$, and $K_{d}$, the motion of the servo motor can better follow the expectation, which is necessary for the control of the BBCPS. The requirement for parameter tuning is that the servo motor can achieve fast and stable motion and keep its motion error at about 0.1%. In response to this requirement, we experimentally determined the optimal $K_{p}$, $K_{i}$ and $K_{d}$ (the specific tuning process is described in Section 6.2.1).

### 5.2. Binocular Interest-Point Extraction Method

The frequency-tuned salient point detection algorithm is a classical method that analyzes an image from a frequency perspective to identify salient points in the image [34]. In this algorithm, the image needs to be Gaussian smoothed. Then, according to Equation (15), the saliency value of each pixel is calculated, and the pixel with the largest saliency value is the salient point.

(15) $$S\left(p\right)={\left\|I_{\mu }-I(p)\right\|}_{2},$$

where $I_{\mu }$ represents the average feature of the image in Lab color space, I(p) denotes the feature of the pixel point p in Lab color space, and S(p) refers to the saliency value of the pixel point p. However, this algorithm can only determine the salient point in a single image. What we need to extract are interest points in binocular images.

When humans perceive a scene, one eye plays a leading role [48]. Inspired by this, we used the left camera as the leading eye, and proposed a binocular interest-point extraction method based on frequency-tuned and template-matching algorithms [49]. The flow of this method is shown in Algorithm 1.

The detailed description of Algorithm 1 is as follows. After inputting the left camera image $I_{l}$, the right camera image $I_{r}$, the image width W, and the template image width $w_{t}$, the frequency-tuned salient point detection algorithm is first used. The interest point $p_{l}=[x_{l},\;y_{l}]$ in the image $I_{l}$ is obtained. With the point $p_{l}$ as the center, the template image $I_{t}$ with a size of $w_{t}$ × $w_{t}$ is determined. A template-matching algorithm is used to match the corresponding interest point in the image $I_{r}$. Since the vertical visual field angles of the left and right cameras are consistent in the calibrated BBCPS, a matching algorithm through local search is performed to improve the speed. The starting location of the sliding window [$x_{s\min}$, $y_{s\min}$] in the image $I_{r}$ is defined as [0, $y_{l}-\;2w_{t}$], the ending location [$x_{s\max}$, $y_{s\max}$] is [$W-w_{t}$, $y_{l}+\;2w_{t}$], and the sliding step *d* is 1. The similarity *S* between the sliding window and the template image at each position in the traversal interval is calculated using the mean square error. We find the maximum *S* and record the corresponding location of the sliding window [$x_{s}$, $y_{s}$]. Finally, the interest point $p_{r}$ in the right camera image is obtained.

**Algorithm 1:** Binocular interest-point extraction method based on frequency-tuned and template-matching algorithms.**Input:** $I_{l}$, $I_{r}$, W pixels, $w_{t}$ pixels**Output**: $p_{l}$, $p_{r}$Obtain $p_{l}$ in $I_{l}$ using the frequency-tuned algorithm [34]Extract a template image $I_{t}$, with a size of $w_{t}$ × $w_{t}$, centered on $p_{l}$
**for** $y_{s}$ $\leftarrow y_{s\min}$ to $y_{s\max}$ step *d* do   **for** $x_{s}$ ← $x_{s\min}$ to $x_{s\max}$ step *d* do          Compute *S* and record [*S*, $x_{s}$, $y_{s}$]   
**end for**

**end for**
Find the maximum *S* and record the corresponding $x_{s}$ and $y_{s}$
Obtain $p_{r}\;=\;[x_{s}\;+\;0.5w_{t},\;y_{s}\;+\;0.5w_{t}]$


### 5.3. Binocular Cooperative Motion Strategy

**Figure 9:**
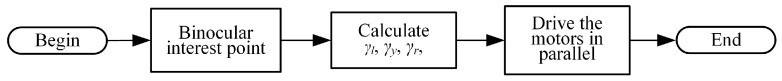
Bionic binocular cooperative motion strategy.

**Figure 10:**
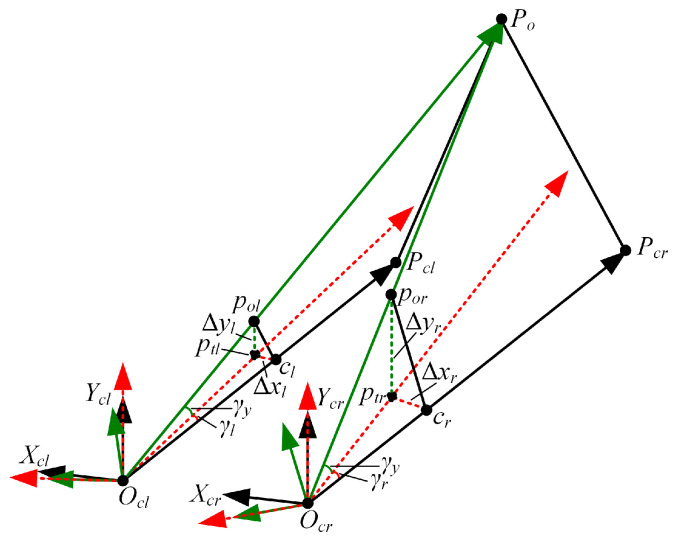
The rotation angles of the three servo motors.

To imitate the movement mechanism of the gaze function, we developed a binocular cooperative motion strategy. The implementation process of the strategy is shown in Figure 9. First, we used Algorithm 1 to extract the binocular interest point. Afterward, the rotation angle of the left servo motor $\gamma_{l}$, the rotation angle of the right servo motor $\gamma_{r}$, and the rotation angle of the upper servo motor $\gamma_{y}$ were calculated. The principle of calculating the rotation angles of the three servo motors is shown in Figure 10.

In this paper, we use $O_{cl}X_{cl}Y_{cl}Z_{cl}$ to define the left camera coordinate frame and $O_{cr}X_{cr}Y_{cr}Z_{cr}$ to describe the right camera coordinate frame. The points $O_{cl}$ and $O_{cr}$ are located at the left and right rotation centers. The 2D points $p_{ol}$ and $p_{or}$ are the projection points of the 3D interest point $P_{o}$ in the left and right images, respectively. $P_{cl}$ and $P_{cr}$ are 3D points located on the optical axes of the left and right cameras. Their corresponding 2D points are the principal point of the left camera $c_{l}$ and the principal point of the right camera $c_{r}$. The difference between $c_{r}$ and $p_{or}$ is denoted by ${∆x}_{r}$ and ${∆y}_{r}$, and the difference between $c_{l}$ and $p_{ol}$ is described by ${∆x}_{l}$ and ${∆y}_{l}$. The rotation angles of the three servo motors can be obtained via Equation (16). The rotation direction is specified in Section 4.2.

(16) $$\gamma_{l}=\arctan\left|\frac{\Delta x_{l}}{f}\right|,\gamma_{r}=\arctan\left|\frac{\Delta x_{r}}{f}\right|,\;\mathrm{and}\;\gamma_{y}=\arctan\left|\frac{\Delta y_{l}+\Delta y_{r}}{2f}\right|.$$

Finally, the calculated rotation angles are sent to the three servo motors at the same time to realize the cooperative motion of the left and right cameras.

## 6. Experiments

In this part, we summarize the simulated and real experiments that were conducted. The simulated experiment was used to verify the superiority of the BBCPD assembled according to the principle of symmetrical distribution around the center compared to a device assembled according to the classical low center of gravity principle. Real experiments were then performed to test the control performance of BBCPS.

### 6.1. Simulation

**Figure 11:**
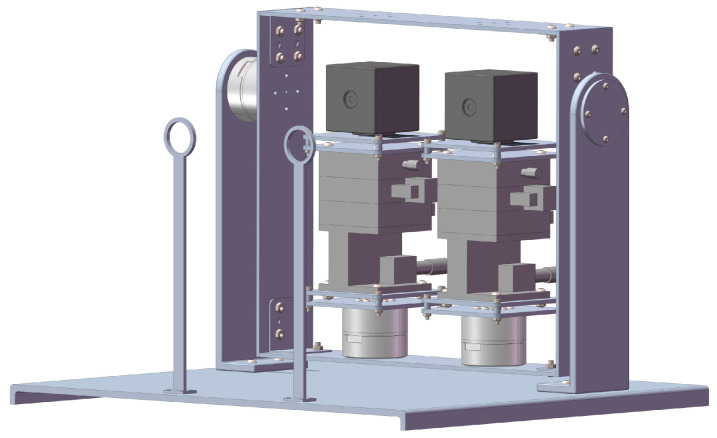
A device assembled according to the classical low center of gravity principle.

**Figure 12:**
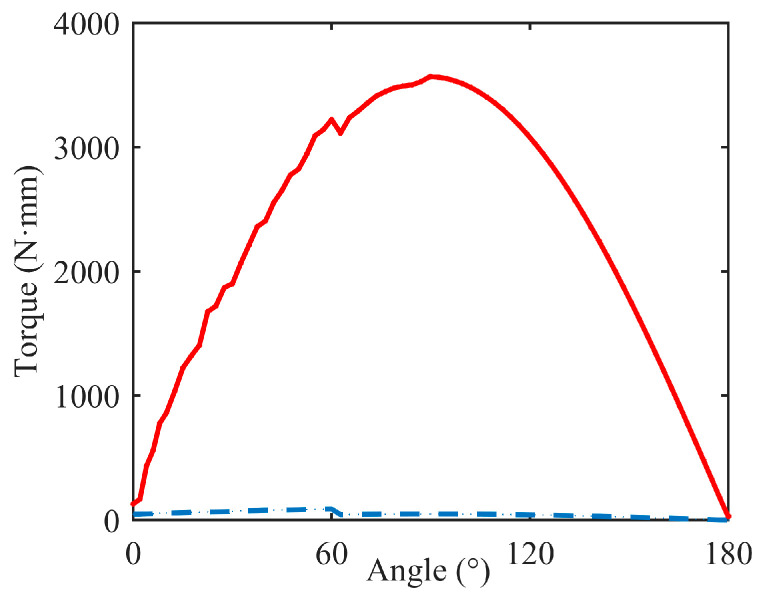
Simulation results, where the blue line represents the BBCPD and the red line represents the device shown in Figure 11.

The simulated experiment was performed to verify that the torque required by the upper servo motor in the BBCPD was less compared with that in the device shown in Figure 11. For this purpose, we used the SolidWorks Motion module to complete the simulation. We imported the 3D models in Figure 3 and Figure 11 into the SolidWorks Motion module and entered the mass of each component. Then, we set the simulation parameters. The speed of the upper servo motor was $\frac{\pi }{3}$ rad·$s^{-1}$, and the acceleration time was 0.5 s. Generalized-alpha stability time-step integration for flexible figures (GSTIFF) [50] is an integral method of variable order and variable step size. This was selected as a type of integrator, suitable for various motion analysis problems. The initial integrator step was 1 × $10^{-4}$, the minimum integrator step was 1 × $10^{-7}$, and the maximum integrator step was 1 × $10^{-3}$. The maximum number of iterations was 25. Afterward, the simulation was carried out. When the plane of the transmission frame was perpendicular to the plane of the base, the rotation angle of the upper servo motor was 0. The simulation results are shown in Figure 12.

As the angle increases, the torques in the device and the BBCPD increase. When the angle was 90°, the torques reached their maximum values. The maximum torques in the BBCPD and the device were 49.5 N·m and 3565.8 N·m, respectively. This showed that the assembly principle of symmetrical distribution around the center can effectively reduce the maximum torque by 98.6%. When the angle was 0°, the torques in the BBCPD and the device were 46.9 N·m and 129.8 N·m, respectively. According to Equation (3), we find that the smaller the torque at the angle of 0°, the smaller the rotational inertia. Therefore, the inertia effect on the braking of the BBCPD is lower than that in the device. In addition, the torque could be effectively reduced by more than 97% in the BBCPD at any angle, which showed that the BBCPD greatly reduces energy consumption.

### 6.2. Real Experiments

Next, we designed a real experiment to validate the control method of the BBCPS. The control method requires the BBCPD to complete initial position calibration. The initial position calibration method requires a known and accurate rotation angle of the servo motor. Therefore, the real experiment was divided into four parts. The first part of the experiment involved tuning the parameters of the PID controller to ensure that the servo motor could move stably and accurately. The second part of the experiment focused on verifying the effectiveness of the initial position calibration method, which lay a foundation for the subsequent experiments. In the third part of the experiments, we aimed to evaluate the accuracy of the proposed binocular interest-point extraction method, which can affect the control performance of the BBCPS. The accuracy of the binocular interest-point extraction method is limited by the accuracy of the template-matching algorithm. Thus, an experiment of template matching for images with viewpoint change was implemented. In the last part of the experiment, we verified the effectiveness of the binocular cooperative perception strategy and analyzed the control performance of the BBCPS.

**Figure 13:**
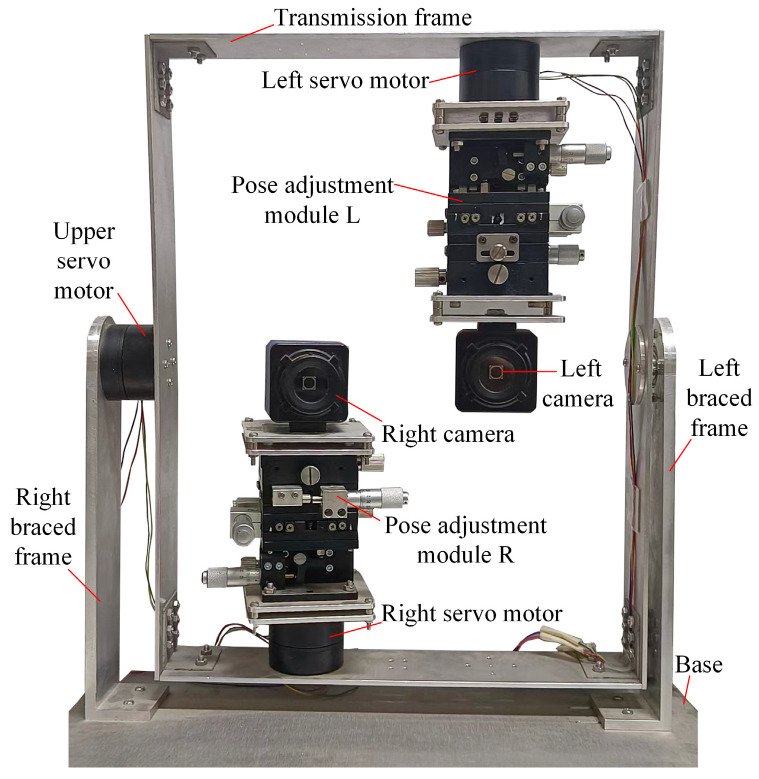
Real BBCPD.

To conduct these experiments, we constructed a real BBCPD, as shown in Figure 13, and corrected the lens distortions of the left and right cameras. The resolutions of the left and right cameras were 640 pixels × 480 pixels, their focal lengths were 532 pixels, and their principal points were [319 pixels, 242 pixels]. The three servo motors used were HT-S-4315, employing the RS485 communication mode. The rotation adjustment accuracy of the pose adjustment module R was 0.01°, and the translation adjustment accuracy was 0.01 mm. The configuration of the pose adjustment module L was the same as that of the pose adjustment module R.

#### 6.2.1. PID Controller Parameter Tuning

**Figure 14:**
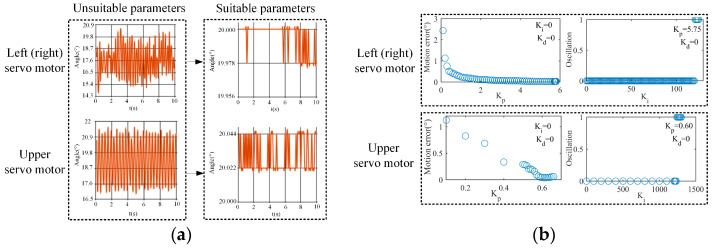
Parameter tuning. (**a**) Steady-state response curve of the motion, (**b**) process of tuning $K_{p}$, $K_{i}$, and $K_{d}$.

In this part of our work, we used the approach of controlling variables to tune $K_{p}$, $K_{i}$, and $K_{d}$ one by one to achieve fast and stable motion of the servo motor and maintain its motion error at about 0.1%. Taking the accuracy motion control of the servo motor to achieve 20° ± 0.022° as an example, during the tuning process, we judged whether $K_{p}$, $K_{i}$, and $K_{d}$ met the control requirement according to the motion steady-state response curve shown in Figure 14a.

The parameter tuning process is shown in Figure 14b. First, we tuned $K_{p}$ while setting $K_{i}$ and $K_{d}$ to zero. The increase in $K_{p}$ can reduce the servo motor’s motion error, but excessively large $K_{p}$ may lead to overshooting, causing an increase in the motor’s motion error. Therefore, we gradually increased $K_{p}$ from 0 until overshooting occurred, and then we determined $K_{p}$. Subsequently, while keeping $K_{p}$ and $K_{d}$ constant, we tuned $K_{i}$. The increase in $K_{i}$ can improve the response speed of the servo motor, but excessive $K_{i}$ may introduce oscillations. Thus, $K_{i}$ was gradually increased from 0 until oscillations occurred, and the maximum $K_{i}$ without oscillation was taken. During this process, when the fluctuation range of the motion steady-state response curve exceeded 0.022°, we regarded that oscillations occurred and were recorded as 1. $K_{d}$ is used to suppress overshooting and oscillation, but an excessively large $K_{d}$ can slow the response speed. Since $K_{p}$ did not cause overshooting, $K_{d}$ could be as small as possible to ensure the response speed. At that time, the parameter tuning was finished. For the right and left servo motors, $K_{p}$ was 5.75, $K_{i}$ was 113, and $K_{d}$ was 0.5. For the upper servo motor, $K_{p}$ was 0.60, $K_{i}$ was 1240, and $K_{d}$ was 0.4.

#### 6.2.2. Initial Position Calibration of the BBCPD

**Figure 15:**
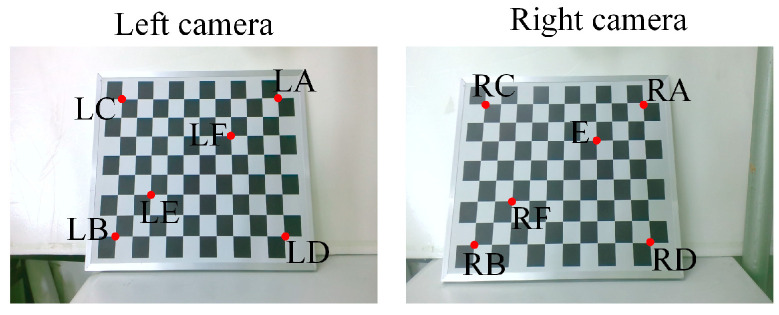
Selected corners.

The aim of this part of our work was to verify the effectiveness of the initial position calibration method for the BBCPD in Figure 13. First, the camera pose calibration and adjustment method was verified. According to the principle outlined in Section 4.1, we used an 8 × 11 checkerboard, shown in Figure 15, to calibrate the rotation parameters $\theta_{x}$, $\theta_{y}$, and $\theta_{z}$. The calibration results are shown in Table 1. According to the calibrated parameters, the pose adjustment module R and the pose adjustment module L were adjusted to ensure that the photosensitive planes of the right and left two cameras were parallel. On this basis, we realized the calibration of the translation parameters $l_{x}$, $l_{y}$, and $l_{z}$. The calibration results are shown in Table 1. Adjustments to the pose adjustment modules were then performed according to the calibration results to ensure that the optical centers of the two cameras were located at the rotation centers. The adjustments of the two camera poses were completed.

Since the adjustment of the camera rotation pose is the basis for the calibration of the translation parameters, it is only necessary to verify the effectiveness of the calibration and adjustment of the camera translation parameters. We used the checkerboard to perform six groups of validation experiments for each of the left and right cameras. As shown in Figure 15, images of the checkerboard were taken by the left and right cameras, respectively. We selected three pairs of corners in the image taken by the left camera (LA-LB, LC-LD, LE-LF) and three pairs of corners in the image taken by the right camera (RA-RB, RC-RD, RE-RF) as the experimental objects. The first group of experiments for the left camera was completed by using the camera to capture an image of the checkerboard after rotating 3° around the horizontal axis and 2° around the vertical axis. Similarly, the first group of experiments for the right camera was completed by using the camera to capture an image of the checkerboard after rotating 3° around the horizontal axis and 2° around the vertical axis. In the second to sixth groups of experiments for the left camera, rotation angles around the horizontal axis were {5°, 8°, 12°, 15°, 20°} and around the vertical axis were {3°, 5°, 9°, 13°, 16°}. For the second to sixth groups of experiments with the right camera, rotation angles around the horizontal axis were {5°, 8°, 12°, 15°, 20°} and around the vertical axis were {3°, 5°, 9°, 13°, 16°}.

**Figure 16:**
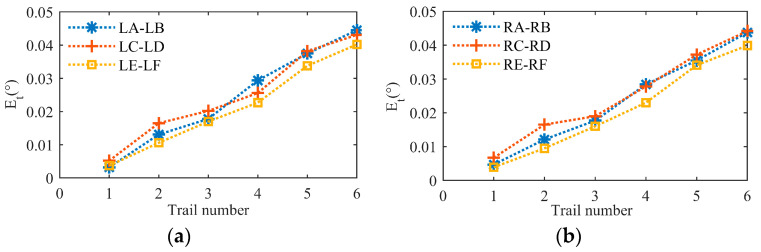
Calibration errors after camera pose adjustment. (**a**) Left camera, (**b**) right camera.

$E_{t}$ is defined as an evaluation index. The specific expression for $E_{t}$ is presented in Equation (17).

(17) $$E_{t}=\left|\left(\varphi_{i0}-\varphi_{j0})-(\varphi_{i1}-\varphi_{j1}\right)\right|,$$

where $\varphi_{i0}$ represents the angle between the light passing through a corner *i* and the optical axis before the camera rotates, $\varphi_{i1}$ is the angle between the light passing through a corner *i* and the optical axis after the camera rotates, $\varphi_{j0}$ refers to the angle between the light passing through corner *j* and the optical axis before the camera rotates, and $\varphi_{j1}$ is the angle between the light passing through corner *j* and the optical axis after the camera rotates. If the optical center of the camera coincides with the rotation center, $E_{t}$ is equal to 0 regardless of the degree of camera rotation. Through the Harris corner extraction algorithm, $E_{t}$ in each group was calculated. The experimental results are shown in Figure 16.

$E_{t}$ was not equal to 0. This indicated that the camera center did not completely coincide with the rotation center. Further observation revealed that the maximum $E_{t}$ did not exceed 0.05°. When converting $E_{t}$ into pixel information based on the known camera focal length, the pixel error generated by an error of 0.05° was less than 1. Considering the corner detection error, the servo motor motion error, the adjustment error of the pose adjustment module, and other factors, we surmised that an error below 1 pixel can imply the effectiveness of the camera pose calibration and adjustment method. Additionally, by combining the rotation angle of the camera in each group of experiments, we observed that as the rotation angle increased, $E_{t}$ also increased. The reason for this is that the optical centers of the left and right cameras do not perfectly coincide with the rotation centers, and the selected corner points are not on the same depth plane. Corners at different depths generate different motion disparities after the camera moves. With an increase in the rotation angle, the difference in motion disparity between two corners at different depths increases, leading to the increase in $E_{t}$.

**Figure 17:**
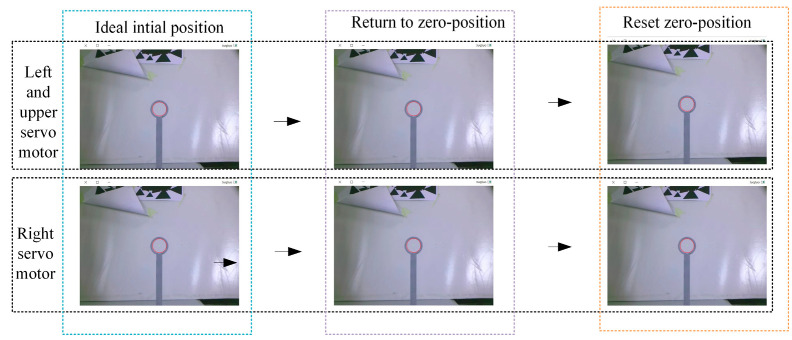
The process of calibration and adjustment of the servo motor zero-position.

Next, the calibration and adjustment method for the servo motor zero-position was verified. According to the calibration principle of Section 4.2, the process of the calibration and adjustment for the three servo motors is shown in Figure 17. First, the pixel coordinates of the ring center $p_{c}$ were determined through Hough circle detection, when the BBCPD was in the ideal initial position. The three servo motors returned to their zero-positions. We detected the pixel coordinates of the ring center $p_{0}$. According to Equation (13), the calibrated angles for the left, upper, and right servo motors were determined as 26.2°, 8.5°, and 19.6°, respectively. Based on the calibrated angles, the zero-positions of the three servo motors were reset.

To validate the adjustment effectiveness of the servo motor zero-position, we conducted six groups of experiments on the three servo motors following the “rotating-zeroing” procedure. In each group of experiments, the rotations of the three servo motors were the same. The rotation angles of the three servo motors in the six groups of experiments were {3°, 5°, 6°, 15°, 10°, 13°}. We defined the Euclidean distance between $p_{c}$ and the pixel coordinates of the ring center detected from the image taken by the left camera when the servo motors returned to the zero-positions as $E_{l}$. Similarly, for the right camera, this was denoted as $E_{r}$. The experimental results are listed in Table 2. After multiple motions of the servo motors, the average $E_{r}$ and the average $E_{l}$ were both less than 1 pixel. The adjustment of the servo motor zero-position was feasible, with $E_{r}$ and $E_{l}$ having little effect on the subsequent control performance. Further analysis was not conducted.

#### 6.2.3. Template Matching for Images with Viewpoint Change

**Figure 18:**
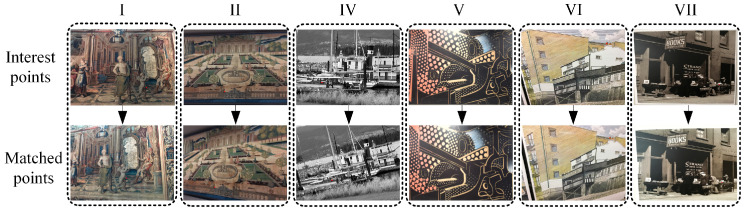
Template-matching experiment.

To test the accuracy of our template-matching algorithm, six groups of experiments were carried out. As shown in Figure 18, experimental images were selected from the publicly available dataset HPatches [51], featuring images with various viewpoints. We used the FT algorithm to extract the pixel coordinates of an interest point in an image. Subsequently, a template with a size of 60 pixels × 60 pixels was used to match this interest point in another image, and the matched point was recorded as $p_{i}$. The dataset provides a mapping relationship between images, enabling the calculation of the position of this interest point in another image, which is considered the ground truth $p_{r}$. We define $E_{m}$ as an evaluation index, as shown in Equation (18).

(18) $$E_{m}={\left\|p_{i}-p_{r}\right\|}_{2}.$$

The results of the experiments are presented in Table 3. Considering the existence of estimation errors in the mapping matrix, it is generally accepted that a condition for correct matching is $E_{m}$ within 3 pixels. Except for the fifth group of experiments, the $E_{m}$ values of all other experiments were less than 3 pixels. This indicates that our template-matching algorithm is suitable for matching images with different viewpoints. Combined with the images in Figure 18, we analyzed the fifth group of experiments. It can be observed that the images in the fifth group of experiments have larger variations in viewpoint compared to the other groups. This suggests that our template-matching algorithm may not be suitable for scenes with significant viewpoint changes, which is a direction for our future research. For other groups where images had smaller variations in viewpoint, the average $E_{m}$ was less than 0.8 pixels. This indicates that our template-matching algorithm performs well when the scene viewpoint does not vary significantly, with an error of around 1 pixel.

#### 6.2.4. Binocular Cooperative Perception

**Figure 19:**
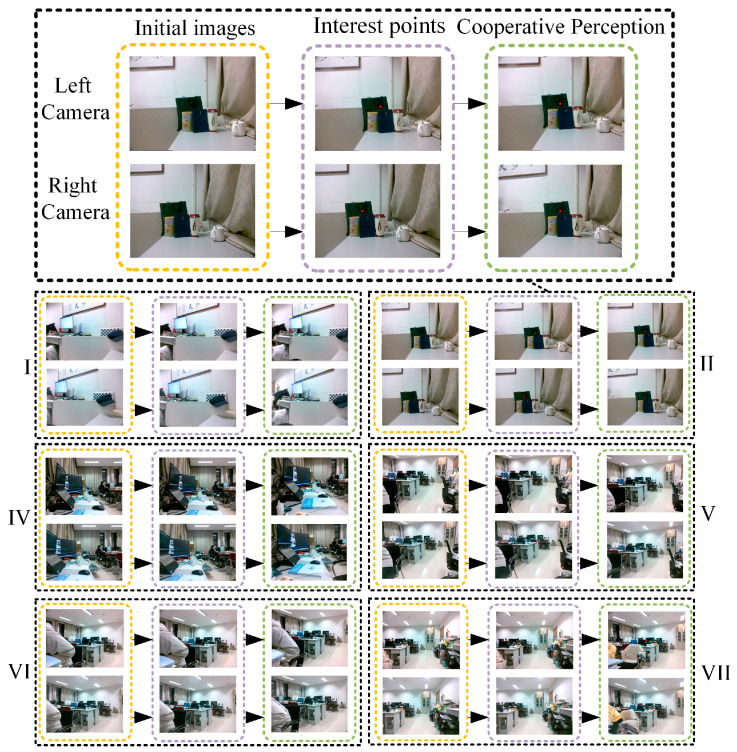
Binocular cooperative perception experiment.

In this part of our work, we conducted six groups of experiments to verify our binocular cooperative perception method. The experimental process is shown in Figure 19. Taking the second group of experiments as an example, the left and right cameras took images at the initial position. Next, the pixel coordinates of the interest points in the left and right cameras were extracted using the binocular interest-point extraction method outlined in Section 5.2. In this process, the size of a template image was set to 30 pixels × 30 pixels. Finally, the motion angles of the three servo motors were calculated using Equation (16), and then the three servo motors were driven in parallel to set the gaze on the interest point. To avoid randomness, we conducted another five groups of experiments following the same steps.

**Figure 20:**
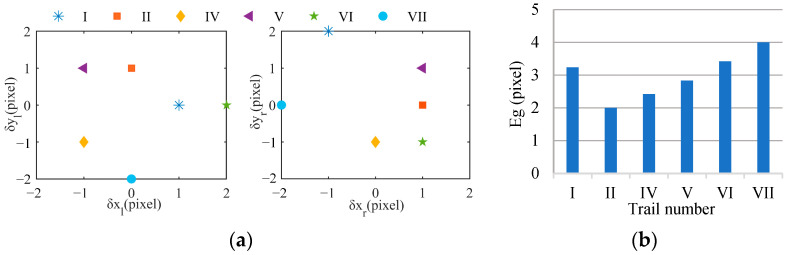
Errors of binocular cooperative perception. (**a**) Error distribution, (**b**) gaze error.

The gaze error $E_{g}$ is defined as an evaluation index, as expressed in Equation (19).

(19) $$E_{g}={\left\|p_{gl}-c_{l}\right\|}_{2}+{\left\|p_{gr}-c_{r}\right\|}_{2},$$

where $p_{gl}$ represents the pixel coordinates of the interest point in the left image after gazing, $p_{gr}$ comprises the pixel coordinates of the interest point in the left image after gazing, and $c_{l}$ and $c_{r}$ are the principal points of the left and right cameras. The experimental results are shown in Figure 20.

Figure 20a illustrates the distribution of the gaze error. The symbols ${\delta x}_{r}$ and ${\delta y}_{r}$ are the difference between $c_{r}$ and $p_{gr}$, and the meanings of ${\delta x}_{l}$ and ${\delta y}_{l}$ are the same as those of ${\delta x}_{r}$ and ${\delta y}_{r}$. Figure 20b shows the $E_{g}$ of each experiment. We found that the absolute values of ${\delta x}_{l}$, ${\delta y}_{l}$, ${\delta x}_{r}$, and ${\delta y}_{r}$ in each group of experiments were less than 2 pixels, and the average $E_{g}$ was less than 3 pixels. This indicates that the perceived interest point basically coincides with the principal point, and it confirms the effectiveness of our control method. The error is attributed to the static error of the servo motor, the template-matching error, and the initial position calibration error. The static error of the servo motor can lead the actual motion angles of the servo motors to deviate from the calculated $\gamma_{l}$, $\gamma_{r}$, and $\gamma_{y}$. The template-matching error results in the matched interest point in the right image deviating from the true corresponding interest point. This means that the calculated $\gamma_{y}$ is not the angle needed in order for the interest point completely coincide with the principal point. The initial position calibration error introduces the interference of the depth information of the interest point. The difference motion disparity results in a difference between the calculated angle and the angle needed for the interest point to completely coincide with the principal point.

Further observation found that ${\delta x}_{l}$, ${\delta y}_{l}$, ${\delta x}_{r}$, and ${\delta y}_{r}$ fluctuated in the six groups of experiments. The reason is that, in different groups, the static error of the servo motor varies, the template-matching error differs, and the influence degree of the initial position calibration error is also different. In each group of experiments, ${\delta x}_{l}$, ${\delta y}_{l}$, ${\delta x}_{r}$, and ${\delta y}_{r}$ also exhibited fluctuations. These were because the static errors of the left and right servo motors may differ and the camera pose adjustment errors for the left and right cameras may also vary. We also noted that $E_{g}$ was the smallest in the second group of experiments, at 2 pixels, while $E_{g}$ was the largest in the sixth group of experiments, at 4 pixels. Our analysis indicates that the viewpoint change in the images affects $E_{g}$. With an increase in the viewpoint change, the rotation angle increases, and the static error of the servo motor also increases. This can lead to a larger difference between the calculated angle and the actual motion angle, potentially resulting in an increase in $E_{g}$. Moreover, an increase in the viewpoint change enlarges the error of the initial position calibration, and then the interest point generates a larger motion disparity. This can lead to a larger difference between the calculated angle and the angle needed for the interest point to completely coincide with the principal point, potentially further increasing $E_{g}$.

## 7. Conclusions

In this study, motivated by the eye gaze mechanism, we designed the flexible BBCPD. The device was assembled according to the principle of symmetrical distribution around the center based on dynamic analysis. The innovative principle offers distinct advantages by enhancing braking performance and reducing energy consumption in comparison to the classic symmetrical low center of gravity principle (as shown in Figure 11). A simulation was conducted to verify the advantages. The results showed that the innovative principle could reduce the torque of the upper servo motor by more than 97%, which leads to a reduction in energy consumption of the BBCPD. The results also demonstrated that the principle could lead the BBCPD to have smaller rotational inertia of the load of the upper servo motor, thus enhancing the braking performance of the BBCPD.

Furthermore, we developed an initial position calibration technique for the BBCPD. Based on the calibration results, the BBCPD, after adjusting the pose adjustment modules and resetting the zero-positions of the servo motors, meets the requirement of the control method. Subsequently, the control method was proposed, where a binocular interest-point extraction method based on frequency-tuned and template-matching algorithms was applied to detect the interest points. Then, we crafted a binocular cooperative motion-control strategy for how servo motors could coordinate their movements and thus set the gaze upon an interest point. Last, real experiments were conducted, and the results showed that the control method of the BBCPS could achieve a gaze error within 3 pixels.

The proposed BBCPS can advance the development of humanoid intelligent perception, with application prospects in fields such as intelligent manufacturing [52,53], human–robot interaction [5], and autonomous driving [54]. However, the gaze accuracy of the BBCPS may constrain its further development. In the future, we aim to reduce gaze errors by optimizing our control algorithm. For instance, by referring to previous research on image matching under viewpoint changes [55,56], we plan to improve the matching algorithm in order to enhance the precision of the binocular interest-point extraction algorithm.

## Figures and Tables

**Table 1:** Camera pose calibration results.

Parameter	$\theta_{x}$ (°)	$\theta_{y}$ (°)	$\theta_{z}$ (°)	$l_{x}$ (mm)	$l_{y}$ (mm)	$l_{z}$ (mm)
Left camera	−3.12	0.89	2.33	15.11	−20.42	16.27
Right camera	−1.56	1.45	−3.42	−9.07	−13.64	15.57

**Table 2:** Calibration errors after servo motor zero-position adjustment.

Trail Number	1	2	3	4	5	6
$E_{l}$ (pixel)	1.0	0.0	0.0	1.4	1.4	1.0
$E_{r}$ (pixel)	0.0	1.0	1.4	1.0	1.0	1.4

**Table 3:** Template-matching results.

Trail Number	1	2	3	4	5	6
$p_{i}$ (pixel)	(610, 594)	(340, 696)	(293, 496)	(325, 561)	(821, 210)	(825, 975)
$p_{r}$ (pixel)	(608, 594)	(339, 696)	(293, 495)	(325, 561)	(822, 213)	(825, 975)
$E_{m}$ (pixel)	2.0	1.0	1.0	0.0	3.2	0.0

## Data Availability

The data presented in this study are available on request from the corresponding author. The data are not publicly available to protect the privacy of the subjects.
